# Do incidental positive emotions induce more optimistic expectations of decision outcomes? An empirical study from the perspective of event‐related potential

**DOI:** 10.1002/brb3.3491

**Published:** 2024-04-19

**Authors:** Ruinan Zhao, Liqing Zhou

**Affiliations:** ^1^ Jing Hengyi School of Education Hangzhou Normal University Hangzhou Zhejiang China; ^2^ Think Tank Alliance of China Education Modernization Research and Evaluation Center Hangzhou Normal University Hangzhou Zhejiang China

**Keywords:** FRN, incidental emotions, P2, risky decision‐making

## Abstract

**Introduction:**

Previous research has found that incidental emotions of different valences (positive/negative/neutral) influence risky decision‐making. However, the mechanism of their influence on psychological expectations of decision outcomes remains unclear.

**Methods:**

We explored the effects of different incidental emotions on the behavioral, psychological, and electrophysiological responses of individuals in risky decision‐making through a money gambling task using a one‐way (emotion type: positive, negative, neutral emotions) between‐subjects experimental design.

**Results:**

Individuals with positive emotions had significantly greater risk‐seeking rates than those with negative emotions during the decision selection phase (*p* < .01). In the feedback stage of decision outcomes, individuals showed stronger perceptions of uncertainty in the decision environment under gain and loss feedback compared with neutral feedback, as evidenced by a more positive P2 component (i.e., the second positive component of an event‐related potential). Positive emotions produced greater than expected outcome bias than neutral emotions, as evidenced by a more negative FRN component (i.e., the feedback‐related negativity component).

**Conclusion:**

Our results suggest that positive emotions increase individuals’ psychological expectations of decision outcomes. This study provides new empirical insights to understand the influence of incidental emotions on risky decision outcome expectations.

## Graphical abstract

Starting from the study of the mechanism of the influence of chance emotions on risky decisions, this study aims to explain the influence of chance emotions on outcome expectations, choice preferences, and cognitive processing in risky decisions from the perspective of EIC theory by improving the previous paradigm of risky decision‐making tasks. We found that episodic emotions are injected into decision‐making and induce psychological expectations consistent with current emotions, which provides preliminary electrophysiological evidence for the mechanism of the influence of episodic emotions on risky decision outcome expectations. Investigating this mechanism can help clarify individual risk preferences and cognitive processing under episodic emotions, adjust the psychological expectation of decision outcomes in time, and help optimize the decision‐maker's choice in risky decision‐making.

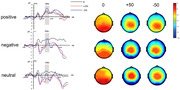

## 1 INTRODUCTION

### 1.1 Background of the study

According to Herbert Alexander Simon (1955), the famous Nobel Prize winner in economics, “decision‐making is the heart of management.” Human beings cannot live without making decisions. Decisions can be classified into deterministic and uncertain decisions according to their certainty (Kahneman, 2003; Zhang & Wang, 2022). Deterministic decisions are those that have only one alternative, the outcome of the choice is known, and the probability of its occurrence is 1. For example, close your eyes to a ball in an opaque bag full of red balls. The color of the ball can only be red. Uncertain decisions have two or more choices and can be classified as risky decisions and fuzzy decisions according to the measurability of the occurrence probability of a particular outcome (Bylund, 2021). In risky decisions, the probability of occurrence of each choice outcome is known (e.g., a gamble on the roll of a dice). In contrast, fuzzy decisions have an uncertain probability of occurrence of each outcome (e.g., a change in stock price). The vagueness of information in fuzzy decisions limits one's ability to make optimal decisions, making it more difficult to decide. The probability of each decision option is known only in risky decisions, enabling people to make optimal decisions by evaluating the options. In the case of risky decisions, researchers can study and intervene in the decision‐makers’ behavior and discover their perceptions. Consequently, risky decision‐making has received widespread attention in the field of decision‐making research.

Previous studies on risky decision propensities have differentiated risk propensities by setting different bet sizes in risky gambling tasks: options with larger stakes represent risk seeking, while options with smaller stakes represent risk avoidance (Turnbull et al., 2003; Zhang & Wang, 2022). However, all options in this paradigm possess a level of riskiness; choosing any bet implies taking a risk and thus cannot better represent the risk propensity in decision‐making. The present study distinguished between risk‐seeking and risk‐avoiding decision tendencies by simulating a money gambling task in which participants were given the choice of flipping in each round, with the choice of flipping implying a gain or loss and the choice of not flipping implying neither risk nor gain or loss, and the mathematical expectation of the two choices was equal. The influence of incidental emotions on risky decision preferences was further explored based on previous studies.

Early studies on decision problems involved the gain and loss framework. Framing effect: When faced with different representations of the same performance, decision‐makers will have different risk preference choices, even make opposite choices (Luo et al., 2022). According to Tversky and Kahneman's (1981) prospect theory, framing affects risk preferences in decision‐making, with decision‐makers preferring risk avoidance in a gain framework but preferring risks in a loss framework. However, Li and Fu (2018) suggested that when individuals made decisions based on experience, information about gains or losses in the outcome of the previous decision did not significantly affect the decision‐maker's next decision, and that extreme gains or losses could have a greater impact on the decision‐maker. In addition, emotion is a factor that influences the framing effect. Individuals in a positive mood tend to be persuaded by positively framed messages, whereas those in a negative mood tend to adopt negatively framed messages (Tong et al., 2008). Against this background, the present study analyzed the effects of emotions and feedback on individual risky decisions by examining the physiological responses during the feedback stage of decision outcomes using electroencephalography (EEG). We further explored the cognitive processing of individuals influenced by incidental emotions during the feedback stage of decision‐making.

### 1.2 The current status of research

Emotions that influence decision‐making can be classified as integral or incidental emotions, based on their relevance to the decision. Integral emotions are emotions generated by the current decision (e.g., an emotion generated by the fear of stock loss when buying stocks), whereas incidental emotions are usually not related to the decision but occur when making the decision (e.g., people who are in a happy mood make the decision to forgive others more easily) (Zhao et al., 2016). This study focused on the influence of incidental emotions on risky decisions.

Prior research has focused on evoking participants’ emotions through visual and auditory stimuli, such as listening to music and watching videos (e.g., Yang et al., 2020). However, asking participants to recall specific scenes or watch videos can consume their cognitive resources and generate additional variables, which, in turn, affects their performance in decision‐making tasks. Therefore, the present study used picture arousal to manipulate participants’ emotions and classified incidental emotions as positive, negative, or neutral based on emotional valence. This type of emotion manipulation is less affected by additional variables and consumes less cognitive resources than when asking individuals to recall something (Shi et al., 2021).

In previous studies on the influence of emotions on decision‐making, the emotion‐imbued choice (EIC) model has suggested that an individual's choice is influenced by the emotions felt at the time of decision‐making and that current emotions may influence the decision by changing the expectation of the decision's outcome. For example, a state of sadness may cause individuals to downplay the reward associated with a decision (Lerner et al., 2015). Motivations contained in current emotions are often used to explain risk preferences in decision‐making, with one study finding that angry emotions produced approach motivations that make individuals willing to take on more risk in decision‐making (Yang et al., 2018).

Generally speaking, in previous studies, decision expectation is often measured directly before decision‐making or cognitive processing in decision selection stage, lacking posterior analysis in decision feedback stage (Wiese et al., 2022). We reflect the influence of emotion on risk decision expectation through ERP component analysis in decision feedback stage, which provides empirical basis for posterior analysis of decision expectation. The present study attempted to provide new empirical insights into the influence of emotions on decision expectations.

### 1.3 Research hypotheses

The present study used event‐related potential (ERP), a technique that measures human brain activity with high temporal resolution, to assess decision‐making. The ERP not only reflects the activity evoked by sensorimotor processes, but also shows a stable temporal relationship with definable events (Markley, 2015; Rocha et al., 2021). ERPs are now widely used in human decision‐making studies, and the present study focused on two ERP components related to decision‐making: the second positive (P2) component and the feedback‐related negativity (FRN) component.

P2 is an early positive component that peaks at around 200 ms after stimulus presentation and is mainly located near the central frontal region of the brain (Wang et al., 2015). The P2 component is associated with attentional selection and early cognitive processing in decision‐making (Chen et al., 2020). P2 is also an early component that is more sensitive to mental conflict (Rey‐Mermet et al., 2019). Overall, the P2 component reflects the processing of psychological conflict (Rey‐Mermet et al., 2019). Feedback on uncertain decision outcomes implies investing more attentional resources, controlling processing, and accepting more mental conflict. Therefore, the following hypothesis was proposed:

H1: *Gain and loss feedback will induce more positive P2 volatility than neutral feedback*.

The time window of FRN is approximately 250−350 ms after feedback stimulus presentation (Yang et al., 2020). Its wave size reflects the magnitude of deviation between the feedback outcome and expectation, and the larger the deviation of the outcome from expectation, the larger the FRN wave amplitude (Li et al., 2020; Walentowska et al., 2019). We believe that positive emotions cause individuals to be overly optimistic about the outcome of decisions, leading to greater deviations between expected and actual outcomes. Therefore, the following hypothesis was proposed:

H2: *Positive emotion group elicited more negative FRN components than neutral emotion group*.

Based on evidence that monetary losses induce more negative FRN than monetary gains (Bandyopadhyay et al., 2019), the following hypothesis was proposed:

H3: *Loss feedback induces more negative FRN than gain feedback*.

## 2 MATERIALS AND METHODS

### 2.1 Participants

Based on a previous study (Zhao et al., 2016), this study used G*power 3.1.9.4 (Erdfelder et al., 1996) to calculate the sample size. A total sample size of at least 58 participants is required to achieve power of 80% at a significance level of α = 0.05 and moderate effect size of f = 0.25. A total of 83 undergraduate and graduate students, including 28 men and 55 women, participated in the study. The age range was 17−28 years, with a mean age of 20.9 years. The participants were randomly assigned to the positive emotion (*n* = 28), negative emotion (*n* = 28), and neutral emotion (*n* = 27) groups. All participants were right‐handed, had no history of traumatic brain injury or psychiatric disorder, had normal vision or corrected vision, and did not experience any recent major life event. The study has been approved by the local ethics committee and each participant will receive a certain amount of money at the end of the study.

### 2.2 Experimental design

This study used a 3 (emotion type: positive, negative, neutral emotions) × 3 (feedback type: +50, −50, 0) between‐within design, where emotion type was a between‐subjects variable and feedback type was a within‐subjects variable. The dependent variables were decision reaction time, risk‐seeking rate, and EEG components (P2, FRN).

### 2.3 Materials and procedures

In this study, we measured the decision reaction time and risk‐seeking rate of subjects by using self‐made money gambling task, and measured ERP components of P2 and P3 by EEG equipment. In addition, some scales were used to measure the participants’ traits. The influence of additional variables is controlled by the factors measured by the scale. The numerical breadth task from the Wechsler Adult Intelligence Scale, Third Edition (WAIS III) was used to measure working memory in this study. Previous research has shown that multiple cognitive abilities can predict decision‐making ability (Skagerlund et al., 2021). Therefore, the present study used the Integrated Wisdom Scale, which contains multiple cognitive abilities, to measure the overall wisdom of the participants. In addition, individuals’ working memory and narcissistic traits have been found to influence their decision‐making ability (Byrne & Worthy, 2013; Hinson et al., 2003). Thus, the present study used the Narcissism Scale and Digit Breadth Task to measure the relationship between narcissistic traits and working memory, controlling for factors that may affect the dependent variable. Correlation analysis of the test results revealed that the Narcissism Scale, Digital Breadth Task, and Integrative Intelligence Scale scores were not significantly correlated with the dependent variables in this study; therefore, these factors were not included as covariates in the final statistical analysis.

Participants entered the laboratory, and the formal experimental session began after they signed informed consent and their scalp was prepared for the EEG experiment. First, the participants sat in a comfortable position on a chair in front of the computer, with their heads leaning on a stand 0.5 m away from the front of the computer screen, and listened to the experimental rules explained by the experimental leader before viewing 20 start‐up pictures of the corresponding emotion group (positive/negative/neutral), with each picture presented 2 times for 2000 ms. The purpose of playing twice is to enhance the memory of the subject for the picture, so as to enhance the effect of eliciting emotion through the memory of the picture. The emotion evoking pictures used in the experiment were selected from the Chinese Affective Picture System (CAPS) (Bai et al., 2005). After the pictures were presented, the participants were asked to complete a questionnaire, which consisted of three questions measuring the individual's emotional arousal in the positive, negative, and neutral emotion dimensions, to test the validity of each emotion initiation. Their answers were scored using a seven‐point Likert scale (Wang et al. 2017; Yang et al. 2020). On a scale of 1 to 7, 1 = least happy/sad/calm, and 7 = most happy/sad/calm.

**FIGURE 1:**
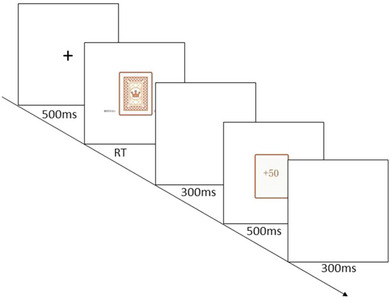
Money gambling task process.

The participants were then asked to complete a money gambling task, and before the task began, they were told that if they flipped a card, they would be presented with a feedback result of +50 or −50, representing their gain or loss for the round, respectively. Participants were told that they needed to maximize their own benefits from the task, and that this correlated with their reward, which was higher on top of the base reward for the higher benefits from the task. The feedback results of “+50,” “−50,” and “0” were defined as gain, loss, and neutral feedback, respectively. Each participant was required to complete 200 rounds of choices, and the numerical results of each round would be cumulated; the larger the cumulative number, the higher the amount of experimental reward received by the participant. The cumulative gains in the decision‐making process were not presented on the screen but were accumulated in the computer background and informed to the participants at the end of the experiment. Participants entered the formal gambling task 200 times after completing five exercises. Participants’ behavioral and EEG data were recorded during the task. In each round of the gambling task, a “+” gaze point lasting 500 ms was presented in front of the computer screen, followed by the back of a deck of cards. If the participant chose to flip the cards, they had to press the A button; otherwise, they had to press the L button. The meaning of the two buttons was balanced within the group. The participant was presented with a blank screen for 300 ms after the key selection, followed by the feedback number “+50,” “−50,” or “0.” After the feedback, a blank screen was presented for 300 ms, and the next round of the task was performed. The experimental flow is shown in Figure 1.

After the decision‐making task, a break was taken to fill out the Integrative Intelligence Inventory (Fu & Wang, 2020) and Narcissism Inventory (NPI‐16), and then the numerical breadth task test used to measure working memory was completed.

### 2.4 EEG recording and analysis

In this study, a 64‐conductor electrode cap extended by the international 10−20 system was used. The reference electrode was located at the midpoint of FPz and Fz on the head during EEG recording, and the ground electrode was located at the midpoint of Cz and Pz. The data were analyzed offline using zero reference, with a filtered bandpass of 0.05−100 Hz, a sampling rate of 1000 Hz/conductor, and an impedance between all electrodes and the scalp of less than 5 KΩ. The data were analyzed offline by independent component analysis to remove artifacts such as electrooculography, head movement, and electrocardiography. The ERP waveforms were digitally filtered at 40 Hz and 0.1 Hz. The EEG analysis time interval was chosen to be −200 to 800 ms. The 200 ms before the stimulus event was used as the baseline for correction. Trials with wave amplitudes greater than ± 100 eve were excluded, and the corresponding ERP data were obtained by superimposing the corresponding trials according to the experimental conditions and stimuli. In the end, the number of valid times stacked for each participant in this study was no less than 98% of the total number of times tested for each experimental condition. There was no significant difference in the number of valid trials under different experimental conditions.

The P2 component of the decision phase and the FRN component of the feedback phase were selected for analysis. P2 is a positive component that peaks at approximately 200 ms after stimulus presentation and is distributed near the central frontal area of the brain (Peng et al., 2021). By analyzing the overall waveform characteristics of the ERP during the decision feedback phase, the P2 component was selected for statistical analysis by selecting the largest peak within 140−170 ms. P2, as an ERP component with earlier time window, is usually calculated by superposing the peak amplitude in time window, while FRN, as an ERP component with later time window, is usually calculated by superposing the mean amplitude in time window. The source of the FRN was localized near the cingulate gyrus region and had the largest component at the midline site of the brain, with the negative component appearing at approximately 250−350 ms after the feedback stimulus presentation (Gehring & Willoughby, 2002; Wang et al., 2017). Therefore, FRN was selected for the statistical analysis of the mean wave amplitude within 250−350 ms. In preprocessing, nine electrode points (F3, F3, Fz, F4, C3, Cz, C4, P3, Pz, P4) were selected for calculation, according to a previous study (Yeung & Sanfey, 2004) and the topographic distribution of each component. The P2 and FRN component wave amplitudes were found to be the largest at the FCz electrode point. Therefore, we selected the electrode point FCz where the P2 and FRN components reached the maximum for further statistical analysis.

## 3 RESULTS

### 3.1 Emotion priming validity test

**FIGURE 2:**
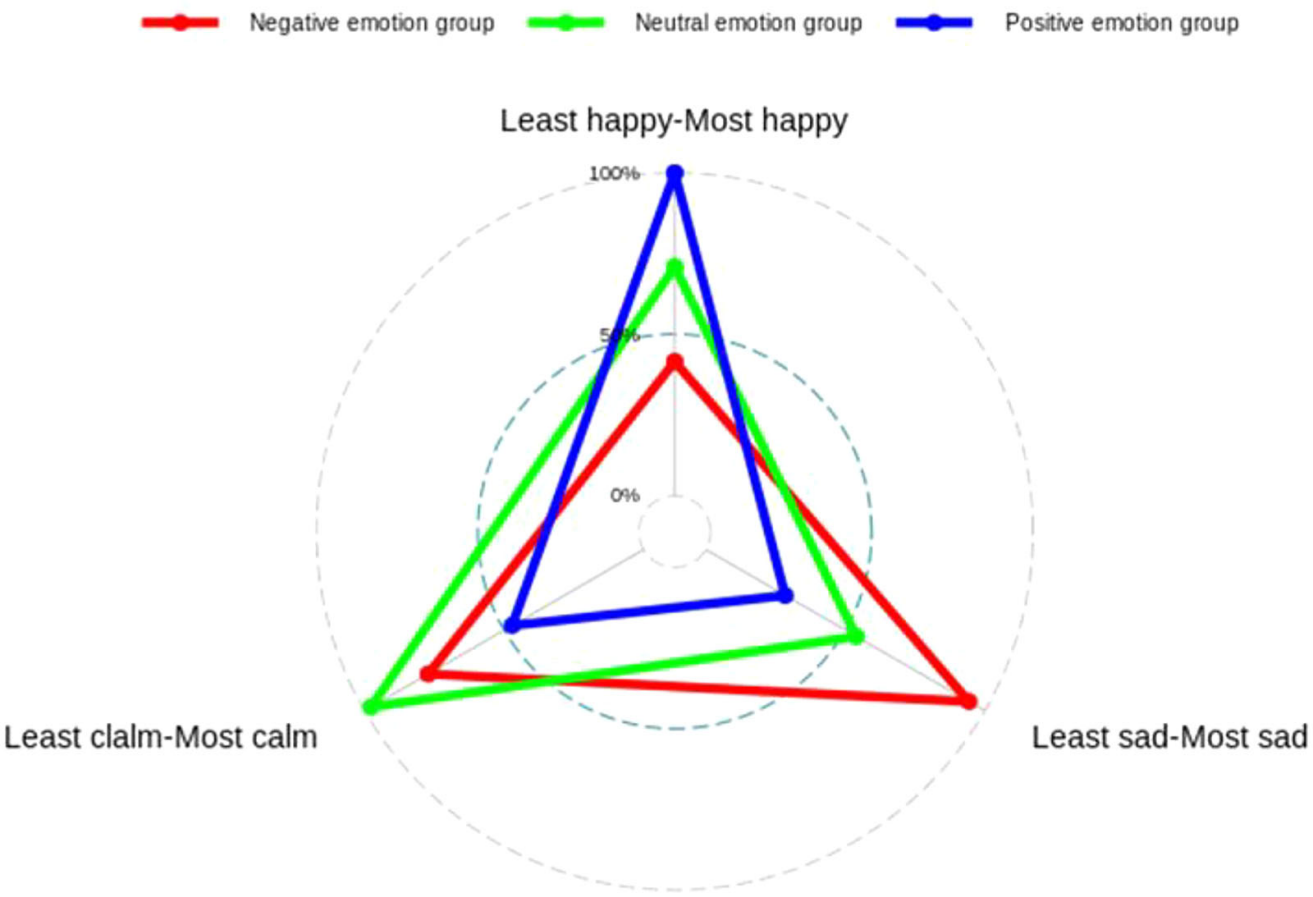
The result of Emotion priming validity test.

A one‐way ANOVA on the results of the ratings of emotion initiation revealed that the differences in emotion type were significant in all three rating dimensions. Post hoc multiple comparisons revealed that in the positive rating dimension, the ratings of the three groups were significantly different (*F*(2,80) = 125.72, *p *< .0001, ${\eta_{p}}^{2}\;$= 0.76). The ratings of the positive emotion group (4.39 ± 0.14) were significantly greater than those of the negative emotion group (1.82 ± 0.10) and the neutral emotion group (3.11 ± 0.10). In the negative dimension, the scores of the three groups were significantly different (*F*(2,80) = 144.47, *p *< .0001, ${\eta_{p}}^{2}\;$= 0.78). The scores of the negative emotion group (4.14 ± 0.12) were significantly greater than those of the neutral mood group (2.37 ± 0.15). In the neutral dimension, there was a significant difference between the three groups (*F*(2,80) = 35.32, *p *< .0001, ${\eta_{p}}^{2}\;$= 0.47). The neutral emotion group scored significantly higher (4.29 ± 0.13) than the positive emotion (3.39 ± 0.24) and negative emotion (2.07 ± 0.18) groups. The results suggest that emotion initiation is effective (Table 1; Figure 2).

**TABLE 1:** Score results of positive, negative and neutral emotion groups.

	Positive rating dimension	Negative rating dimension	Neutral scoring dimension
Positive emotion group	4.39±0.14	1.25±0.08	2.07±0.18
Negative emotion group	1.82±0.10	4.14±0.12	3.39±0.24
Neutral emotion group	3.11±0.10	2.37±0.15	4.29±0.13

### 3.2 Behavioral outcomes

The two main behavioral indicators analyzed in this study were decision reaction time and risk‐seeking rate. Decision reaction time is the time taken by the participants to make key decisions after they view the presentation of the flip interface, which reflects the difference in the cognitive and emotional processing levels of decision‐makers under different emotion initiations. Risk‐seeking rate is the proportion of decision‐makers choosing the risk‐seeking (flip) option to the total number of all choices (i.e., risk‐seeking rate = number of flip choices/[number of flip choices + number of no flip choices]).

**FIGURE 3:**
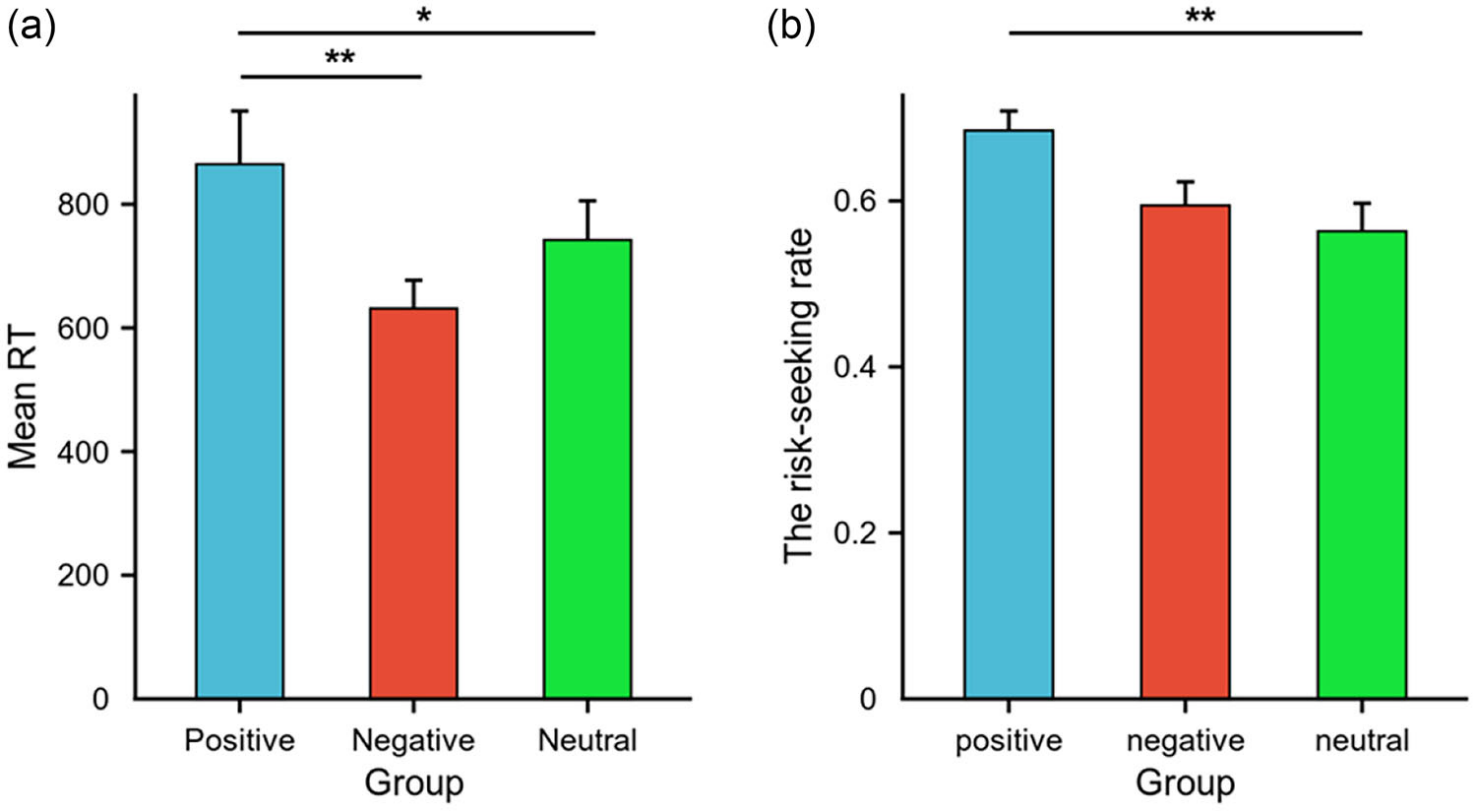
Differences in response and risk‐seeking rates between positive, negative, and neutral emotion groups. (a) The difference in mean RT among different emotional groups. (b) The difference in risk‐seeking rates among different emotional groups.

A one‐way (emotion type: positive, negative, and neutral emotions) between‐subjects ANOVA was conducted for reaction time and risk‐seeking rate for each of the three emotion types. The tests showed that there was a significant difference in decision reaction time between the three emotion types (*F*(2,80) = 3.05, *p *< .05, ${\eta_{p}}^{2}\;$= 0.71). The decision reaction time of individuals in the positive emotion group was greater than that of those in the negative emotion group (positive emotion group: 864.63 ± 455.25 ms; negative emotion group: 631.26 ± 242.13 ms; neutral emotion group: 742.10 ± 329.30 ms). Statistical significance was *p *< .01 for the positive emotion group versus neutral emotion group, *p *< .05 for the positive emotion group versus negative emotion group, and *p* = .45 for the negative emotion group versus neutral emotion group. There was a significant difference in the risk‐seeking rate of decision‐making among the three emotion types (*F*(2,80) = 4.86, *p *< .01, ${\eta_{p}}^{2}\;$= 0.11) (Figure 3a).

The risk‐seeking rate of participants in the positive emotion group was greater than that of those in the negative emotion group and those in the neutral emotion group ([positive emotion group: 0.69 ± 0.03; negative emotion group: 0.59 ± 0.03; neutral emotion group: 0.56 ± 0.03]; [positive emotion group vs. negative emotion group: *p* = .40; negative emotions group vs. neutral emotions group: *p* = .45; positive emotion group vs. neutral emotion group: *p* < .01]) (Figure 3b).

**FIGURE 4:**
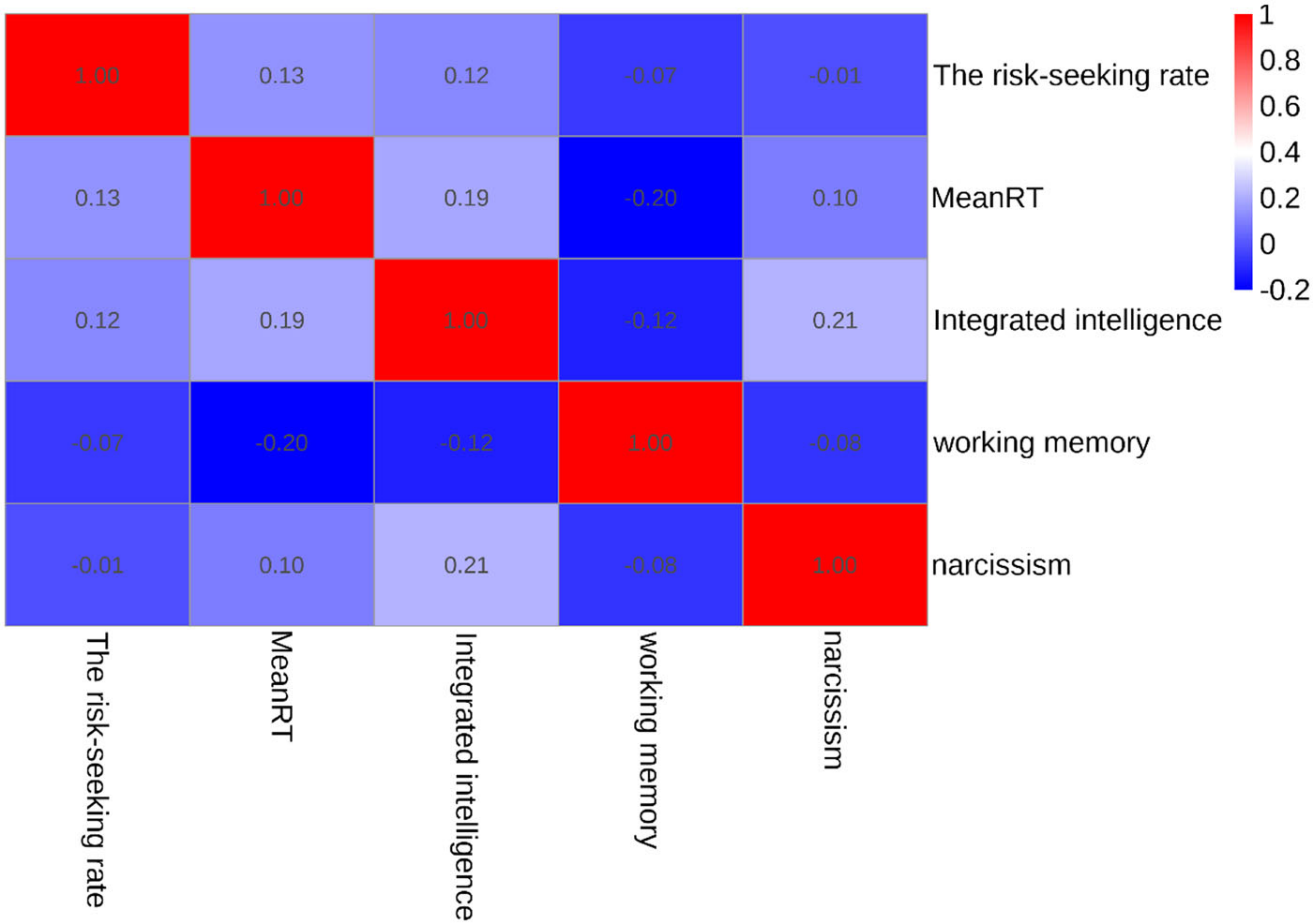
Correlation heat map: Correlation between working memory, integrated intelligence, Narcissism Scale, risk decision response time, risk‐seeking rate.

Pearson product‐difference correlation analysis was performed on the total score, integrated intelligence score, working memory score, decision reaction time, and risk‐seeking rate of Narcissism Scale. The results showed that the correlation coefficients between narcissism score and decision reaction time and risk‐seeking rate were –0.13 and –0.09, respectively, and the correlation coefficients between integrated intelligence score and decision reaction time and risk‐seeking rate were –0.01 and –0.53, respectively. There was no significant correlation between working memory score and decision reaction time and risk‐seeking rate, the correlation coefficients were 0.05 and 0.08, respectively. Narcissistic trait and working memory were not significantly correlated with the dependent variables of this study, indicating that they did not have significant effects on the dependent variables of this study. Therefore, narcissistic trait, working memory, and integrated intelligence were not included as covariates in the subsequent analysis of results as shown in Figure 4.

### 3.3 ERP results

#### 3.3.1 P2

**FIGURE 5:**
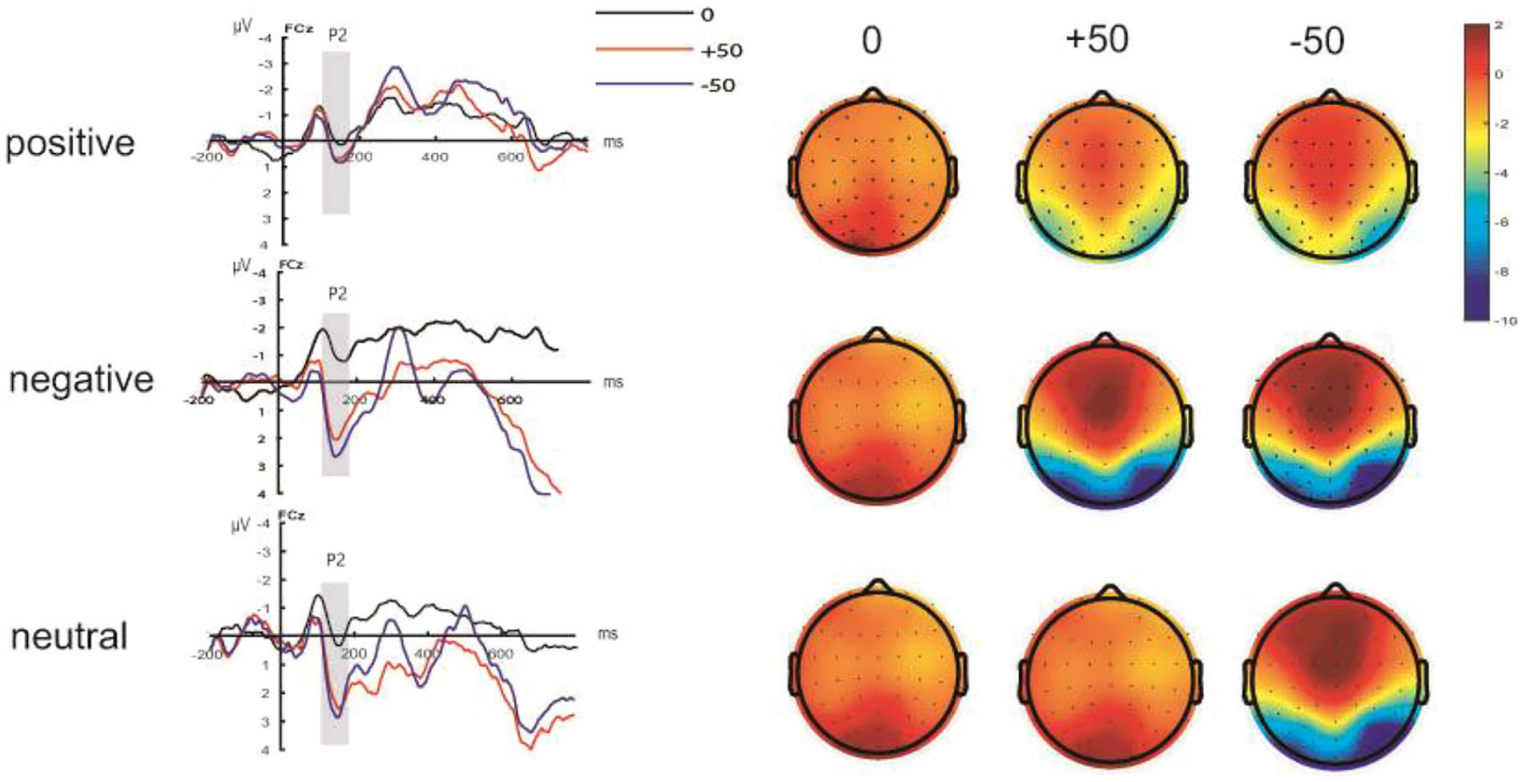
P2 component waveforms and topography in the decision feedback stage.

A 3 (emotion type: positive, negative, neutral) × 3 (feedback type: gain, loss, neutral) repeated‐measures ANOVA was conducted on the peak of the decision feedback phase P2 (140−170 ms). The statistical results showed that the main effect of emotion type was not significant (*F*(2,57) = 2.18, *p *= .12, ${\eta_{p}}^{2}\;$= 0.07) and that of feedback type was significant (*F*(2,114) = 25.88, *p *< .001, ${\eta_{p}}^{2}\;$= 0.31). The pairwise comparison results indicated that the P2 amplitude of gain and loss feedback was significantly larger than that of neutral feedback ([gain feedback: 2.39 ± 0.36 µV; loss feedback: 2.52 ± 0.35 µV; neutral feedback: −0.52 ± 0.4 µV]; [gain feedback vs. neutral feedback: *p *< .001; loss feedback vs. neutral feedback: *p* < .001]). Emotion type and feedback type interaction effects were not significant (*F*(4,114) = 1.08, *p *= .37, ${\eta_{p}}^{2}\;$= 0.04) (Figure 5).

#### 3.3.2 FRN

A 3 (feedback type: gain feedback, loss feedback, neutral feedback) × 3 (emotion type: positive emotion, negative emotion, neutral emotion) two‐way ANOVA on the mean wave amplitude of FRN (250−350 ms) during the feedback phase revealed a significant main effect of emotion type (*F*(2,57) = 4.44, *p *< .05, ${\eta_{p}}^{2}\;$= 0.14).

#### 3.3.3 Post hoc comparison

**FIGURE 6:**
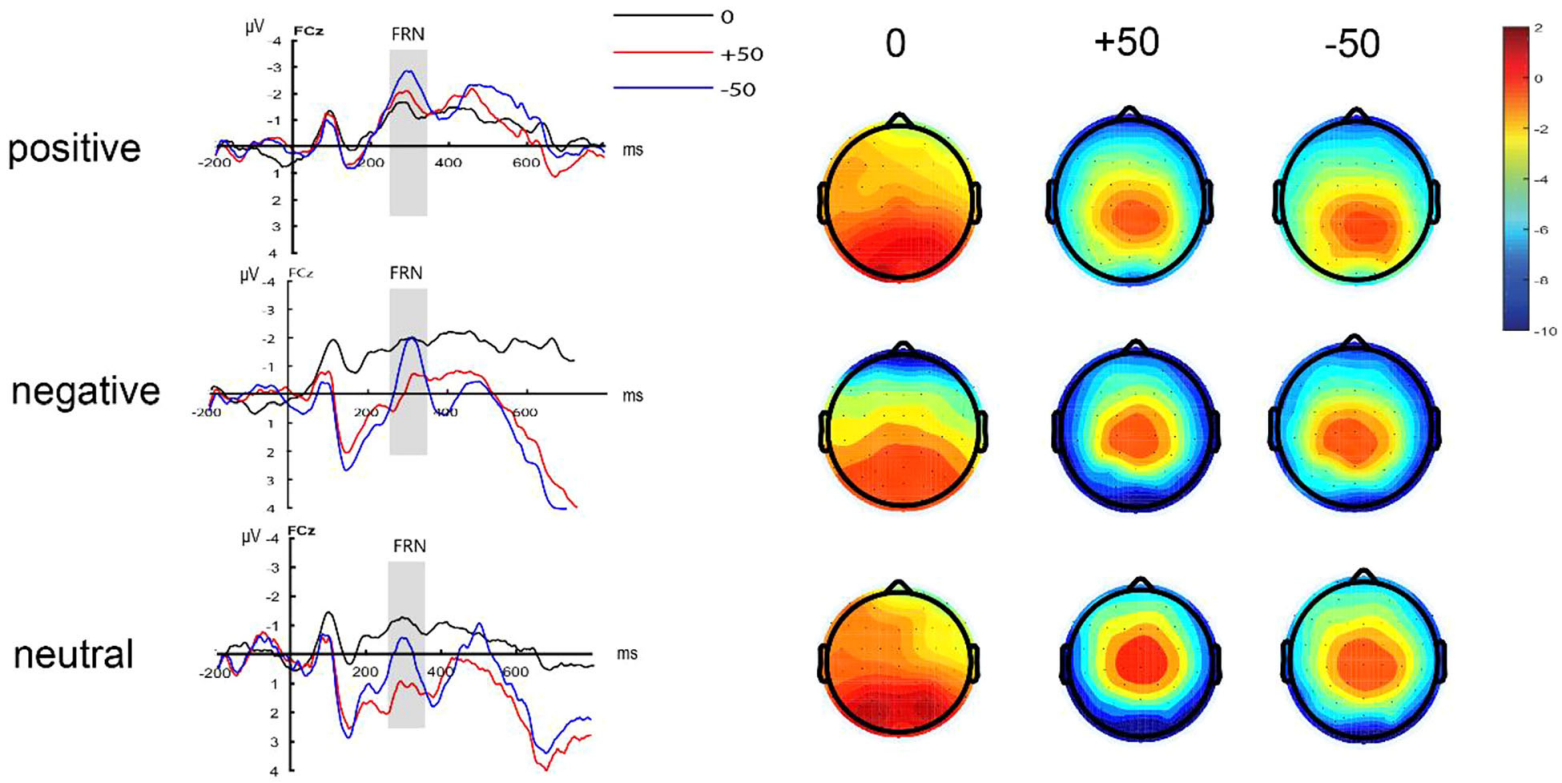
FRN component waveforms and topography in the decision feedback stage.

A post hoc comparison revealed that the mean FRN amplitude in the positive emotion group was significantly smaller than that in the neutral emotion group (positive emotion group: −1.80 ± 0.46 µV, negative emotion group: −1.01 ± 0.46 µV, neutral emotion group: 0.12 ± 0.46 µV, positive emotion group vs. neutral emotion group: *p* < .01, negative emotion group vs. positive emotion group: *p* = .08, positive emotion group vs. neutral emotion group: *p* = .23). The main effect of feedback type was significant (*F*(1.39,79.01) = 3.61, *p *< .05, ${\eta_{p}}^{2}\;$= 0.06). Post hoc pairwise comparisons revealed that the mean FRN volatility for neutral and loss feedback was significantly smaller than that for gain feedback ([gain feedback: −0.20 ± 0.36 µV; loss feedback: −1.12 ± 0.36 µV; neutral feedback: −1.37 ± 0.36 µV]; [gain feedback vs. neutral feedback: *p *< .05; gain feedback vs. loss feedback: *p *< .001; loss feedback vs. neutral feedback: *p *= .62]) (see Figure 6). The interaction between emotion type and feedback type was not significant (*F*(2.77,79.01) = 1.81, *p *= .16, ${\eta_{p}}^{2}\;$= 0.06).

## 4 DISCUSSION

The behavioral results of this study showed that the risk‐seeking rate of decision‐making was significantly greater for participants with positive emotions than for those with negative emotions. This result supports the EIC model to some extent, as participants experiencing positive emotions evaluated the environment for decision‐making more positively and thus dared to try high‐risk high‐reward options. In contrast, participants experiencing negative emotions assessed the decision environment more negatively and chose more conservative options compared with those experiencing positive emotions.

Regarding individual decision reaction time, positive emotions increased individual expectation of outcome, and higher expectation of outcome promoted individual risk‐taking. The longer response time could reflect weighing of options in the face of potential harm despite an emotionally primed motivation for risk. There are two processes operating that are, to some extent, acting oppositely on the final behavioral response. The results showed that the decision reaction time of positive emotion group was significantly higher than that of negative and neutral emotion group. Longer decision response times reflect increased risk. The results also support the EIC theory. Previous research has shown that affective regulation of expected outcomes is specific to positive arousal, not to other emotional states. Positive arousal promotes the redefinition of potential losses as potential gains to safety (Knutson et al., 2004). Previous studies have provided evidence for this process.

The ERP results for the feedback phase of the decision showed that the P2 amplitude of the gain and loss feedback was significantly larger compared with that of the neutral feedback. This result is consistent with previous research (Liu & Dou, 2015), suggesting that P2 reflects the perception of uncertainty in the decision situation. As such, there is more uncertainty under gain and loss feedback compared with neutral feedback. Thus, high risk and uncertainty make gain and loss feedback induce a more positive P2 component.

The mean FRN volatility was significantly greater in the positive emotion group than in the neutral emotion group, suggesting that experiencing positive emotions induced the processing of expected positive outcomes. This finding is consistent with previous theoretical perspectives (Bower, 1981; Lerner et al., 2015). Individuals generate mental expectations in decision‐making that are consistent with their current emotions. Positive emotions induce positive expectations of decision outcomes, and individuals are more likely to associate good decision outcomes when in a positive emotional state. Thus, our study provides new empirical insights into the EIC theory. That is to say, positive emotions improve individuals’ subjective expectations of decision results and induce more negative FRN in the feedback stage of decision results. In addition, the FRN component of positive emotions and negative emotions did not reach a statistically significant level, which may be due to the fact that negative emotions include anger and fear pictures. Previous research has found that anger and fear have different effects on decision‐making (Yang et al., 2018). Anger emotions induce approaching motivation, which makes individuals take more risks and thus produce higher outcome expectations, so it shows a larger FRN component. Because of the existence of anger, the FRN difference between positive emotion and negative emotion is not significant.

Plus, the FRN component induced by gain feedback is significantly smaller than that induced by neutral and loss feedback, which indicates that the deviation between individual expectation and outcome under neutral feedback is smaller, which may be caused by the author's decision goal. In this study, participants were asked to make decisions to maximize their own interests, so gain feedback is feedback that meets individual decision goals, and the outcome is more consistent with psychological expectations, thus producing smaller FRN components.

This study investigated the effects of three incidental emotions (positive, negative, and neutral) on individuals’ risky decisions, and we expected that under the influence of positive emotions, individuals would have more optimistic psychological expectations of decision outcomes. The findings of this study fill a gap in the research on decision‐making. Our study provides an empirical basis for emotion‐imbued choice (EIC) model and has theoretical implications for researchers and academicians in that it expands the literature on the influence of emotions on decision‐making (Elliott et al., 2023; Perach et al., 2021). In risky decision‐making, individuals experiencing positive emotions produce more risk‐seeking behaviors in decision‐making compared with those experiencing negative emotions. This suggests that individuals in environments imbued with high positive emotions and positive feedback need to strengthen safety management to avoid high‐risk behaviors. The study has practical implications for managers; for example, in music festivals, it would be important to do a thorough job of managing and providing safety, security, and the necessary fire‐fighting measures at the site.

Nevertheless, the study had some limitations. First, the influence of emotions on decision‐making varies depending on the participant's age. An FMRI study found that older adults had greater activation in the right anterior‐ventral insula cortex than younger adults when viewing pictures of emotional faces, suggesting that there are differences in the processing of emotional information by individuals of different ages (Fischer et al., 2005; Zhang et al., 2022). The data come from healthy volunteers and cannot be generalized to people with gambling problems or other impulsive or risk‐prone populations. Future research could explore how the influence of emotions on individuals in risky decision‐making varies with age. Future research may explore how the impact of emotions on individual risk‐taking decisions changes with age. We can discuss further the differences between healthy people and gambling addicts. Second, the present study induced emotions in participants before a money‐gambling task and then allowed them to make multiple risky decisions, with the influence of emotions diminishing over time during the process of making multiple decisions. However, this method is applicable to contexts where multiple risky decisions are made after being influenced by incidental emotions. This better simulates the problem that people are faced with multiple decisions after being affected by emotions in the real situation. In contrast, Yang et al. (2018) presented participants with different emotional faces at each decision choice to provide timely emotional arousal to make decisions, which can address the weakening of emotions over time. Moreover, although we use the money gambling task to simulate real‐world decisions, there is still a certain gap with real‐world decisions. Third, this study used a between‐subjects design, and the experimental results were influenced by the individual differences of the participants. This study did not control such variance due to background factors, as no covariates were included in the main analyses. Future studies should control background factors that can influence the relationship between emotions and risky decision‐making. Furthermore, we did not directly measure individual risk preferences, and future studies should do so to control for the effects of individual variability on risk decisions.

## 5 CONCLUSION

Starting from the study of the mechanism of the influence of incidental emotions on risky decisions, this study aims to explain the influence of incidental emotions on outcome expectations, choice preferences, and cognitive processing in risky decisions from the perspective of EIC theory by improving the previous paradigm of risky decision‐making tasks. We found that incidental emotions are injected into decision‐making and induce psychological expectations consistent with current emotions, which provides preliminary electrophysiological evidence for the mechanism of the influence of incidental emotions on risky decision outcome expectations. Investigating this mechanism can help clarify individual risk preferences and cognitive processing under incidental emotions, adjust the psychological expectation of decision outcomes in time, and help optimize the decision‐maker's choice in risky decision‐making.

## AUTHOR CONTRIBUTIONS

**Ruinan Zhao**: Conceptualization; methodology; formal analysis; investigation; writing—original draft. **Liqing Zhou**: Conceptualization; methodology; writing—review and editing; funding acquisition; resources; supervision.

## CONFLICT OF INTEREST STATEMENT

The authors have no conflicts of interest to declare that are relevant to the content of this article.

### INFORMED CONSENT STATEMENT

All participants recruited for this study participated in this study voluntarily after signing an informed consent form for participating in the experiment prior to participation.

### PEER REVIEW

The peer review history for this article is available at https://publons.com/publon/10.1002/brb3.3491.

## Data Availability

The datasets generated during and/or analyzed during the current study are available from the corresponding author on reasonable request.
